# Therapeutic Potential and Challenges of Natural Killer Cells in Treatment of Solid Tumors

**DOI:** 10.3389/fimmu.2015.00202

**Published:** 2015-04-29

**Authors:** Andrea Gras Navarro, Andreas T. Björklund, Martha Chekenya

**Affiliations:** ^1^Department of Biomedicine, University of Bergen, Bergen, Norway; ^2^Karolinska University Hospital, Hematology Center and Karolinska Institute, Stockholm, Sweden

**Keywords:** NK-cell subsets, KIR-HLA interactions, tumor microenvironment, cancer stem cells, clinical application

## Abstract

Natural killer (NK) cells are innate lymphoid cells that hold tremendous potential for effective immunotherapy for a broad range of cancers. Due to the mode of NK cell killing, requiring one-to-one target engagement and site-directed release of cytolytic granules, the therapeutic potential of NK cells has been most extensively explored in hematological malignancies. However, their ability to precisely kill antibody coated cells, cancer stem cells, and genotoxically altered cells, while maintaining tolerance to healthy cells makes them appealing therapeutic effectors for all cancer forms, including metastases. Due to their release of pro-inflammatory cytokines, NK cells may potently reverse the anti-inflammatory tumor microenvironment (TME) and augment adaptive immune responses by promoting differentiation, activation, and/or recruitment of accessory immune cells to sites of malignancy. Nevertheless, integrated and coordinated mechanisms of subversion of NK cell activity against the tumor and its microenvironment exist. Although our understanding of the receptor ligand interactions that regulate NK cell functionality has evolved remarkably, the diversity of ligands and receptors is complex, as is their mechanistic foundations in regulating NK cell function. In this article, we review the literature and highlight how the TME manipulates the NK cell phenotypes, genotypes, and tropism to evade tumor recognition and elimination. We discuss counter strategies that may be adopted to augment the efficacy of NK cell anti-tumor surveillance, the clinical trials that have been undertaken so far in solid malignancies, critically weighing the challenges and opportunities with this approach.

## Introduction

Cancer is a generic group of over 100 diseases unified by fundamental characteristics acquired during their clonal evolution (1), including tumor-promoting inflammation and escape from immune surveillance (2, 3). Increased cancer incidence is associated with immunodeficiency (4, 5) and sustained immunosuppression (6–8). This supports the immunological surveillance hypothesis that postulates that as well as conferring protection from infectious pathogens, the immune system also guards against cancer (9). A prospective study of a large cohort of Japanese inhabitants followed up for 11 years uncovered an association of low natural cytotoxicity of peripheral blood natural killer (NK) cells with approximately 40% increased cancer risk compared to individuals with high cytotoxicity activity (10). This is reinforced by findings of decreased NK-cell activity among relatives of patients diagnosed with familial melanoma (11), implicating NK cells in tumor surveillance. Novel immunotherapies have recently demonstrated unprecedented success in selected cancer types (12, 13). Heightened interest in applicability of NK cells for cancer immunotherapy has been evoked by improved methodologies for their purification, molecular and phenotypic characterization. This progress is a result of a deeper understanding emerging from 40 years of scrutiny of their roles in anti-cancer immunity. In this article, we discuss the current knowledge on how NK cells recognize and kill cancer cells while sparing the normal healthy cells, how the cancer and its associated cells exploit this biology. We discuss the strategies that may be employed to derive effective medicine, the promise and challenges of these approaches for solid malignancies.

## NK Phenotypes and Natural Cytotoxicity Against Solid Tumors

Natural killer cells comprise 10–15% of peripheral blood lymphocytes and classically display a half-life of approximately 7–10 days in circulation (14–16). However, some subpopulations generated by cytokine stimulation or viral infections may display prolonged persistence (17–19). NK cells arise from lineage restricted progenitor cells derived from CD34^+^ common lymphoid progenitors through a process regulated by basic leucine zipper transcription factors, E4-promoter binding protein 4 (E4pb4), Ets1 (20, 21), and the T-box transcription factors, T-bet, Eomes, as well as ID2 (inhibitor of DNA binding). In their absence, fewer numbers of NK cells develop (22–25) and exhibit functional alterations (26). Terminally differentiated NK cells lack phenotypic markers of B and T lymphocytes and are distinguished further by their greater size and cytoplasmic granularity. They express CD56 neural cell adhesion molecule and are thus phenotypically categorized as CD3^-^CD19^-^CD56^+^ innate lymphoid cells (27–29).

Functionally, NK cells can lyse directly virus-infected or transformed cells without prior sensitization (28, 30), mediated by natural cytotoxicity receptors (NCRs) NKp30 (CD337), NKp44 (CD336), and NKp46 (CD335) that belong to the Immunoglobulin superfamily (31). NKp30 is constitutively expressed and recognizes B7-H6 tumor antigens (expressed on leukemia, lymphomas, carcinomas, and melanomas) (32). NKp44 is expressed only on activated human NK cells and binds viral hemagglutinin (HA) and HA-neuraminidase (HN) as well as tumor-associated ligands (33). NKp46 is expressed on both resting and activated NK cells in mice and men and can inhibit growth of tumor metastases in mice (34, 35), although its ligands have yet to be identified. The NK group 2D (NKG2D, CD314) is a lectin-like type 2 transmembrane homodimeric receptor that is constitutively expressed on all NK cells (36). It transduces activating signals upon binding to its ligands major histocompatibility complex (MHC) class 1-related chains A and B (MICA and MICB) and viral UL16 binding proteins (ULBPs). These stress induced-ligands are expressed on tumor, but not healthy cells, as a result of heat shock, viral infection, or genotoxic stress by DNA damage (37). NKG2D ligation induces phosphorylation of YINM motifs on DAP10, allowing recruitment and activation of the p85 subunit of phosphatidylinositol-3-kinase (PI3K) and growth factor receptor-bound protein 2 (GRB2) to trigger NK cytotoxicity (38). We recently demonstrated that NKG2D was highly expressed by glioblastoma (GBM) infiltrating NK cells *in situ* (39). Antibody blockade of NKG2D rescued approximately 50% stress ligand-bearing GBM but not K562 chronic myelogenous leukemia (AML) cells, from lysis by donor NK cells *in vitro* (40). This emphasizes the importance of activation signaling via NKG2D for NK cell cytotoxicity. Indeed, proteolytic cleavage of NKG2D ligands by ADAM 10 and 17 proteases (a disintegrin and metalloproteinase) sheds soluble ligands into serum to circumvent cytotoxicity via NKG2D receptor (41, 42), and is a common aberration in cancer (43). Soluble MICA/B and ULBPs have been detected in sera of patients with diverse solid malignancies (44), where soluble ULBP2 distinguished early stage pancreatic adenocarcinoma from healthy subjects. Elevated ULBP2 could identify melanoma patients at risk for disease progression and was prognostic in patients with early stage B-cell chronic lymphocytic leukemia (45–47). Conversely, others demonstrated that hypoxia induced microRNAs miR-20a, miR-93, and miR-106b downregulated NKG2D ligands on GBM cells as a mechanism of immunological escape (48). Genome wide association studies also identified a MICA-A5.1 allelic variant with a frameshift mutation that results in a truncated protein that is released as a membrane-anchored molecule in exosomes in human papilloma virus induced cervical cancer in a Swedish cohort (49, 50). Another MICA variant, rs23596542, was identified in hepatitis C virus induced hepatocellular carcinomas (HCC) from a Japanese population (51). Both cleaved MICA and exosomal MICA-A5.1 result in high serum levels of soluble MICA that interacts with NKG2D and prevents its interaction with membrane bound ligands. Recently, the GBM derived metabolite, lactate dehydrogenase isoform 5 (LDH5), was demonstrated to upregulate the NKG2D ligands MICA/B and ULBPs on monocytes from healthy individuals *in vitro* and on circulating macrophages from patient derived breast, prostate, and HCC as a further means to subvert NK cell surveillance (52). This would lead to NKG2D receptor downregulation through internalization, degradation, and/or desensitization (53). Ultimately, diminished NK cytotoxicity ensues due to chronic exposure to ligand expressing cells, consistent with the discontinuity theory of immunity (54). A caveat to interpreting causality of soluble ligands in patient sera to attenuated NKG2D receptor levels is the presence of transforming growth factor β (TGFβ) that also diminishes NKG2D, as reported in GBM (55). Another emerging concept coined *split anergy* proposes that NK cell-monocyte/macrophage cross-talk *in vitro* results in anergic NK cells that are not cytotoxic but secrete cytokines that enhance differentiation of cancer stem cells (CSCs) (56). CSCs are minor subpopulations within the tumor capable of self-renewal by asymmetrical cell division to maintain the tumor’s cellular heterogeneity (57). CSCs are resistant to conventional anti-cancer therapy (57, 58) and are proposed to drive malignant progression. Differentiated cells are thought to be more resistant to NK lysis (59, 60), but more responsive to the standard treatment. Thus, NK-cell/macrophage crosstalk may halt malignant progression by directly killing and/or differentiating the CSCs (56). Although largely observed *in vitro*, split anergy may have clinical implications for the impact of NK-cell/macrophage crosstalk in tumor surveillance *in vivo*. Nevertheless, natural cytotoxicity is a tumor-dependent killing mechanism that varies in potency because although NCRs and NKG2D are constitutively expressed on NK cells in most individuals, different tumors variably express tumor-associated antigens and NKG2D ligands.

## NK Subsets

On the basis of the density of CD56, NK cells can be further subclassified as CD56^bright^ subsets (61) that also express high levels of l-selectin (CD62L), an adhesion molecule that mediates interaction with the vascular endothelium (62). CD56^bright^ NK cells account for only 10% of circulating peripheral blood NK cells but represent a dominant phenotype in secondary lymphoid tissues. They constitutively express high affinity heterotrimeric interleukin (IL)-2Rαβγ receptors, maintain long telomeres, and proliferate in response to low concentrations of IL-2 (63, 64). Unlike T cells, NK cells do not secrete IL-2 (65) but CD56^bright^ subsets are denoted by secretion of other cytokines, including interferon-gamma (IFN-γ), tumor necrosis factor-alpha (TNF-α), IL-5, IL-10, and IL-13. Notably, IFN-γ promotes T-helper type 1- (T_h_1) responses that enhance effector functions of both NK cells and cytotoxic T lymphocytes (CTLs) (66, 67), up-regulate class I and class II MHC (68) on antigen presenting cells, as well as costimulatory molecules on macrophages (67). CD56^bright^ subsets express CC-chemokine receptors 7 (CCR7), CCR5, and CXCR3 (69) that allow their preferential recruitment to tumor and inflamed tissues (70, 71). CD56^bright^ NK cells also secrete granulocyte colony-stimulating factor (G-CSF) and granulocyte macrophage colony-stimulating factor (GM-CSF) (67, 72, 73) that promote homing to secondary lymphoid organs through endothelial venules (74).

In contrast, the majority (approximately 90%) of NK cells in steady state peripheral blood are CD56^dim^, and are considered to differentiate from the CD56^bright^ CD94/NKG2A^+^ subsets through transitional loss of CD94/NKG2A and CD62L. Acquisition of CD57 and killer immunoglobulin like receptors (KIRs) denotes their terminal differentiation to CD56^dim^ CD57^+^CD62L^-^CD94/NKG2A^-^ KIR^+^ mature NK cells. The CD56^dim^ subsets are more cytotoxic against target cells compared to the CD56^bright^ subsets and proliferate in response to IL-2 and IL-15 stimulation through signaling via their heterodimeric IL-2Rβγ/IL-15Rα. CD56^dim^ CD57^+^ subsets exhibit replicative senescence as indicated by shortened telomeres and diminished proliferation in response to stimulation with cytokine combinations *ex vivo* (75, 76). CD56^dim^ subsets secrete low IFN-γ, even after activation with IL-2, or combination IL-15/IL-21. They lack CCR7 but do express CXCR1, CXCR2, and low density CXCR3, as well as CX3C chemokine receptors 1 (CX3CR1^high^). This traditional designation of CD56^dim^ as “potent killers” and CD56^bright^ subsets as “cytokine producers” might be oversimplified, as both subsets can perform either function when appropriately stimulated (77). NK cells dynamically adjust their phenotypes in response to the changing cytokine concentrations, ligand density, and cell types present in their microenvironment. Thus, it is debated whether the phenotypic subsets represent distinct maturation stages that are also functionally independent subpopulations, regardless of age, diurnal fluctuations, and microenvironments in diseases states, such as cancer (78). If subset characteristics change dynamically depending on their microenvironment, challenges for selecting *a priori* suitable subsets for anti-cancer therapy will be inevitable. All human NK cell subsets express a range of other adhesion molecules, including CD2, CD44, VLA-5 α chain (CD49e), lymphocyte function associated antigen (LFA-1), and intracellular adhesion molecule-1 (ICAM-1). Their density vary and thus impact trafficking patterns to various tissues and ability to form functional immunological synapses during immune responses (71). Both CD56^bright^ and CD56^dim^ NK cell subsets secrete chemokines such as monocyte chemotactic protein-1 (MCP-1, CCL2), macrophage inflammatory protein 1-α (MIP1-α, CCL3), macrophage inflammatory protein 1-β (MIP1-β, CCL4), and regulated on activation normal T cell expressed and secreted (RANTES, CCL5) (72). These chemokines further recruit, activate, and enhance antigen presentation of other immune cells at sites of inflammation, required for full-fledged immune responses (79, 80).

## Trafficking to the Solid Tumor Microenvironment

**Figure 1:**
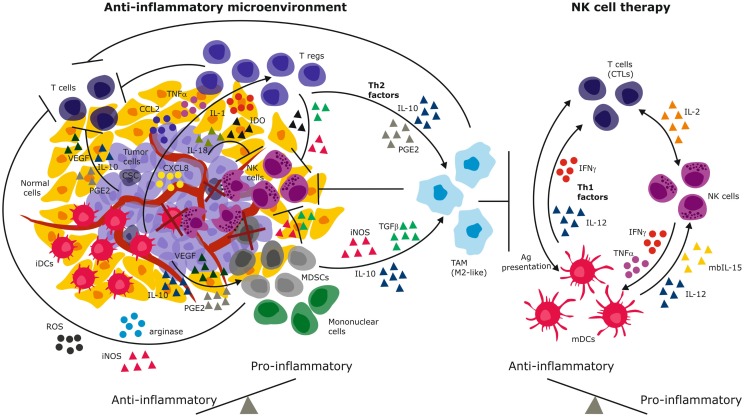
**Immune suppressive mechanisms of the solid tumor microenvironment**. Tumor-derived immunosuppressive mechanisms include secreted cytokines and chemokines that recruit and sustain immature and suppressive immune cells. These in turn secrete Th2 factors that propagate the cycle to create a chronic anti-inflammatory environment that promotes and predominates malignant progression (left). NK cell therapy has the potential to reverse the anti-inflammation to engender dominant pro-inflammatory signaling through secretion of IFN-γ, Th1 cytokines, and activation of both innate and adaptive immune cells (right). By elimination of tumor cells and immature suppressive cells (e.g., iDCs, MDSCs, cancer stem cells), NK cells have the potential to reverse the anti-inflammatory signals and eliminate tumor progression. iDCs, immature dendritic cells; mDCs, mature dendritic cells; MDSCs, myeloid derived suppressor cells; PGE2, prostaglandin E-2; IDO, indoleamine-2,3-dioxygenase; VEGF, vascular endothelial growth factor; TGFβ, transforming growth factor-beta; TNF-α, tumor necrosis factor-alpha; IFN-γ, interferon-gamma; IL, interleukin; iNOS, inducible nitric oxide synthase; ROS, reactive oxygen species; CTLs, cytotoxic T lymphocytes (CTLs); Tregs, regulatory T lymphocytes; CSC, cancer stem cells.

The microenvironment of solid tumors poses a formidable challenge to NK cell efficacy due to chronic immune suppressive signals that select for tumor cells with altered immunogenicity, while simultaneously hindering both infiltration and activation of NK cells at tumor nests (81). The tumor secretes TGFβ (82, 83) vascular endothelial growth factor (VEGF), prostaglandin E2 (PGE2), and IL-10 that suppress T cell proliferation and cytotoxic responses (84) (Figure 1). These factors also downregulate class I MHC, skew dendritic cells toward an immature, tolerogenic, immature phenotype (85). TGFβ and PGE2 generate a highly heterogeneous population of myeloid derived suppressor cells (MDSCs) that comprise granulocytes, macrophages, and dendritic cells arrested at various differentiation stages (86). In response to hypoxia in the tumor microenvironment (TME), MDSCs upregulate arginase activity and inducible nitric oxide synthase (iNOS) (Figure 1) that inhibit T cells through NO signaling (87). This further depletes intracellular l-arginine and enhances production of reactive oxygen species (ROS) that diminish immune surveillance through shedding of NKG2D ligands (87). In addition, MDSCs secrete IL-10 and through membrane bound TGFβ, polarize tumor associated macrophages (TAMs) toward an M2- like anti-inflammatory phenotype (86) (Figure 1). A seminal study demonstrated that a subpopulation of mouse GR-1^+^CD11b^+^F4/80^+^ mononuclear MDSCs expressing the NKG2D ligand RAE-1 did not suppress, but in fact activated NK cells to produce IFN-γ and abolished tumor growth (88). Depletion of this population fueled tumor growth confirming their protective role against cancer. Although this study defines functional plasticity within the highly heterogeneous MDSC pool, the study was performed in a NK cell sensitive, relatively homogeneous mouse tumor. The findings may not easily translate to biochemically complex microenvironments of heterogeneous solid tumors in humans. The function of the RAE-1 expressing GR-1^+^CD11b^+^F4/80^+^ does not easily reconcile findings of the NK tolerizing role of NKG2D ligands on TAMs (39, 52). Others reported that coculture with Gr-1^+^CD11b^+^ MDSCs inhibited cytotoxicity of IL-2 activated NK cells against Yac-1 cells and that this was mediated via membrane bound TGFβ and signaling via signal transducer and activator of transcription-5 (STAT5) (89). Thus, polyclonal MDSCs may exhibit functional plasticity to induce anti-tumor or pro-tumor effects depending on their subset phenotype, tumor type, or microenvironment encountered (Figure 1).

Although CSCs represent a minor population in the TME, they too contribute to immune suppression (90, 91) through STAT3 (92), a signaling hub utilized by diverse cytokines and tumor infiltrating immune cells to markedly suppress their functionality (93). The transcriptional activity of nuclear factor-kappa beta (NFκB) also regulates the function of CSCs and immune effectors at the tumor site (94). Differentiated oral epithelial tumors exhibit higher NFκB activity and are resistant to NK-mediated cytotoxicity compared to their CSC counterparts (59). Inhibition of NFκB in differentiated carcinoma cells, or in non-transformed keratinocytes, increased sensitivity to NK cell lysis (95), implicating NFκB survival signaling in resistance to NK lysis. Indeed, CSCs from oral squamous carcinomas and colorectal cancers with diminished NFκB levels are more sensitive to NK lysis compared to their differentiated counterparts (59, 60, 96). In contrast, NK cells preferentially lyse GBM CSCs despite constitutively activated STAT3 and NFκB signaling (97, 98). Instead, it was reported that low class I MHC and high PVR and Nectin-2 expression levels underlie their sensitivity to NK cells (97, 98). This discrepancy may reflect diversity in oncogenic signaling pathways of CSCs from different tissues.

The tumor secretes chemokines CXCL8, CCL2, IL-1, and TNF-α that recruits macrophages and suppressive CD4^+^CD25^+^CD127^low^FOXP3^+^ Tregs to the tumor site (Figure 1). Tregs have been demonstrated to inhibit NK cells from gastrointestinal stromal tumors (GIST) by competing for IL-2 availability and secrete IL-4, indoleamine-2,3-dioxygenase (IDO). Tregs express membrane bound TGFβ that inhibits NKG2D and NKp30 by cleaving their ligands off malignant cells (99, 100). Tumor-derived IDO promotes production of the immunosuppressive tryptophan catabolite l-kynurenine that interferes with IL-2 induced expression of NKp46 and NKG2D receptors that are required for target cell recognition and killing (101). In contrast to CD4^+^CD25^+^CD127^low^FOXP3^+^ Tregs, we recently identified CD8^+^CD28^–^Foxp3^+^ Tregs that were induced in the GBM microenvironment (39). These Tregs might tolerize tumor infiltrating antigen presenting cells through IL-10 induced immunoglobulin-like transcripts (ILT)-2, ILT3, ILT4, and decreased expression of CD40, CD80, and CD86 costimulatory molecules (39). Decreased Tregs have been associated with improved NK cell activity in GIST, melanoma, and GBM after treatment with Imatinib, and DC exosomes (102) or vaccination (103), underscoring the clinical impact of these suppressive cells. Melanoma and colorectal tumor associated fibroblasts could inhibit NK cell activity through cell–cell contact *in vitro* (104, 105) and through secretion of PGE2 or IDO that abrogated IL-2 induced NKp44, DNAM-1, and NKp30 upregulation (106). Thus, targeting IDO in cervical cancer increased NK cell accumulation at the tumor site and inhibited growth (101).

Indeed, increased infiltration of NK cells into tumor lesions of diverse histological types, such as GBM (107), solid metastases (108) lung, gastric, colorectal, as well as head and neck cancers, have been associated with good prognosis (70, 109–112). In gastric cancer, increased NK cell infiltration correlated with reduced invasion, reduced lymph nodes metastases, and improved outcome. A caveat to these early studies, however, was the utilization of single staining immunohistochemistry against CD56 or CD57 markers that are also expressed by CD8^+^ T cells (107) and some tumor types (113). Moreover, CD57 is only expressed on a subset of CD56^dim^ mature NK cells (75, 76). Thus, the degree of NK cell infiltration in tumors is difficult to ascertain from these studies, as both overestimation and under representation of the figures is possible. Staining for NKp46 that is expressed by all NK cells revealed that renal cell carcinomas (RCC) and GIST had substantial NK cell infiltration (114, 115). However, NK cells infiltrating the RCC were not cytotoxic and upregulated CD94/NKG2A inhibitory receptor. NK cells infiltrating the GIST tumors ostensibly expressed the immunosuppressive NKp30c isoform whose surface levels were also prognostic in these patients (114, 115). Other studies reported scarce infiltration of HCC and RCC by NK cells. The NK cells were functionally anergic as indicated by diminished NKp30, DNAM-1 expression, reduced degranulation or stayed in the stroma, and did not contact the tumor cells (104). Extracellular matrix and tumor cell derived gangliosides (sialic acid glycoproteins), GM2, and GM3 have been demonstrated to diminish NK cell cytotoxicity through contact inhibition to targets, and abrogation of IL-2 dependent proliferation (116). Furthermore, pre-incubation of NK cells with GD3 and GM3, or serum from cancer patients containing shed GD3 prior to addition of target cells, strongly inhibited NK cell lysis in a dose dependent manner (117, 118). Again, the effects of serum could be explained through other mechanisms, such as TGFβ. GD3 and various gangliosides are abundantly secreted into the TME of patient GBMs *in situ* (119, 120), potentially providing similar immunosuppressive mechanisms of escape from NK cell lysis. Precise mechanisms regulating NK cell recruitment to tissues are poorly delineated but may depend on both tumor histological type and chemokine profiles. Therefore, several experimental therapies have attempted to augment endogenous NK cell trafficking and activity at tumor nests. CD27^high^ NK cells in mice are orthologs of human CD56^bright^ cells, and in both species, these NK subsets express CXCR3 and are attracted to CXCL9 and CXCL10 chemokines at tumor sites (121, 122). CD56^dim^ NK cells, however, express CX3CR1 and have been detected in breast, lung, and colorectal cancer biopsies. In the latter, the NK cells remained in the stroma and were not in direct contact with the malignant cells despite elevated levels of chemokines typically expressed by both CD56^dim^ and CD56^bright^ subpopulations (123). This might indicate insufficiency of chemokines alone in determining the impact of tumor infiltrating NK cells. Remarkably, the chemoattractant chemerin has been shown to favor infiltration of melanoma by NK cells, reduced MDSCs, and immunosuppressive plasmocytoid DCs (81, 124). Whole genome expression datasets confirmed that chimerin was prognostic of improved outcome in melanoma patients and expression is lost during malignant progression of several solid tumors. In B16 transplantable mouse melanoma, chimerin overexpression or exogenous injection in the context of its chemokine-like receptor 1 diminished tumor growth (124). It is not known whether CD56^dim^ and CD56^bright^ subsets are differentially recruited to the tumor because of their different chemokine receptor profiles. It is possible that CD56^bright^ subsets might be increased in the TME as a consequence of their enhanced proliferative capacity and or propensity for surviving oxidative stress conditions (125) that typify the TME. Both CD56^dim^ and CD56^bright^ subsets may express CD16, a low affinity receptor (FcγRIII) that binds the constant Fc chain of antibody (126), inducing NK cell activation and killing of antibody coated cells through antibody-dependent cellular cytotoxicity (ADCC). Human breast cancer infiltrating CD56^bright^ NK cells expressing low levels of CD16 but heightened expression of activation markers NKp44, CD25, CD69, and NKG2D were reported (127). Similarly, we reported that CD56^dim^ CD16^-^ NK cells that infiltrate patient GBM lesions expressed NKG2D (39). Loss of CD16 on tumor infiltrating NK cells is one mechanism proposed for induction of split anergy (60, 128). Tumor infiltrating NK cells purified by negative selection from ascites fluid from ovarian cancer patients produced less IFN-γ and IL-4, but more IL-10 compared to corresponding peripheral blood NK cells (129). Although the CD56^dim^ CD16^-^ subsets might indicate potential cytotoxicity, their diminished proliferative potential may result in low intra-tumor effector-target ratios, thus reducing their killing efficiency. Attenuated cytokine production and inability to execute ADCC of antibody-coated cells due to lack of CD16 might render their cytotoxicity unsustainable in hostile solid TMEs. Collectively, these studies indicate a consistent characteristic in solid cancers of loss of NK cell-tumor engagement, cleavage of stress ligands, loss of NKG2D receptor and or CD16, thus diminishing tumor recognition, cytotoxicity, and ability to execute ADCC of antibody-coated targets.

## Antibody-Dependent Cellular Cytotoxicity

Indeed, the induction of ADCC may represent the underlying mechanism for the unprecedented success of monoclonal antibodies to enhance tumor cell recognition by the immune system, exemplified by Rituximab against CD20 in lymphoma (130) and Ipilumimab targeting cytotoxic T-lymphocyte antigen-4 (CTLA-4) in metastatic melanoma (12). IgG1 is the most frequently used isotype for humanized antibodies due to serum stability and high affinity for CD16 on NK cells, or CD32 on neutrophils and monocytes/macrophages (FcγRII). The homozygous FcγRIII-158Val and FcγRII-131His allelic polymorphisms result in proteins with higher affinity for IgG1, IgG3, and IgG4. Patients with these polymorphisms demonstrated greater NK cell–mediated ADCC (131–133). ADCC is executed by the lytic granules, perforin, and granzymes A/B, whereas the release of cytokines and chemokines leads to inhibition of cell proliferation and angiogenesis (134, 135). Thus, as well as exerting efficacy through immune checkpoint inhibition, ipilumimab also triggers ADCC and TNF-α mediated killing by NK cells (134). Nivolumimab, an IgG4 antibody against another immune checkpoint molecule, programed death-1 (PD-1), has been combined with Ipilumimab to harness synergism in blocking immune checkpoints and evoke therapeutic benefits greater than either treatment alone (13). Since tumor cells secrete IL-18 that upregulates PD-1 on NK cells, anti-IL-18 neutralizing mAb in combination with Nivolumimab may circumvent tolerization of NK cells. Strong evidence for the anti-cancer efficacy of ADCC was obtained with Rituximab in patients with various lymphoid malignancies of B-cell origin, including follicular and aggressive large B-cell non-Hodgkin’s lymphoma (130). Trastuzumab/Herceptin for treatment of patients with metastatic breast (136) and early stage gastric carcinomas (137), humanized anti-GD2 mAb in melanoma, neuroblastoma, osteosarcoma, and soft tissue sarcoma patients (138–141) induce potent ADCC. As well as allelic polymorphisms in FcγRIII, high antigen expression is required for improved efficacy. A humanized CD16-CD33 BiKE (bispecific killer engager) antibody was demonstrated to strongly activate NK cells against CD33^+^ AML blasts when combined with pre-treatment with an ADAM17 small molecule inhibitor that prevented shedding of CD16 *in vitro* (142). Cetuximab, a chimeric human/mouse antibody targeting epidermal growth factor receptors (EGFR) for treatment of GBM, advanced non-small cell lung cancer (NSCLC), ovarian cancer, and colorectal cancer, induced NK cell mediated ADCC, and extended overall survival by 3 months in head and neck cancer patients (97, 143, 144). EGFR variant III (EGFRvIII) is a tumor specific mutant epitope that is expressed on one-third of GBMs and mediates an aggressive phenotype. Unarmed mAbs against the EGFRvIII showed promise in GBM preclinical studies, but limited efficacy in patients (145). The latter was likely the result of limited penetration of the tumor bed, regrowth of heterogeneous tumor due to antigen loss variants, and or resistance due to redundancy in oncogenic signaling pathways (146, 147). A promising GBM associated antigen is the cell surface chondroitin sulfate proteoglycan NG2/CSPG4 that is also expressed on various cancer forms, including melanomas, leukemia, breast, and sarcomas, but not in normal differentiated cells in the corresponding tissues (73, 148–150). Elevated NG2/CSPG4 enhances tumorigenicity, angiogenesis and migration, as well as conferring resistance to chemotherapy and radiotherapy via distinct mechanisms (119, 151–157). We demonstrated that NG2/CSPG4 is highly expressed by approximately 20–30% of treatment resistant cells and angiogenic vessels in the TME of 50% of all patient GBMs (119, 151–156). Elevated NG2/CSPG4 expression was an independent prognostic biomarker for poor survival. We showed that combination treatment of adoptively transferred NK cells with mAb9.2.27 against NG2/CSPG4 in preclinical models of GBM induced synergistic therapeutic effects through TNF-α, a IFN-γ release, diminished IL-10, IL-6, and IL-1α. Combination NK cells + mAb9.2.27 induced potent ADCC mediated by Fcγ-IIR on microglia/macrophages that resulted in prolonged survival (158, 159). mAb9.2.27 could not induce ADCC by NK cells *proper*, likely because of its IgG2a isotype, known to engage weakly the Fcγ-RIII on NK cells. However, improved chimeric antigen receptor conjugated or humanized bispecific antibody constructs against this antigen have recently been tested in pre-clinical studies with promising results (160, 161). Natural cytotoxicity and ADCC are not the only mechanisms NK cells use to eliminate malignant cells.

## NK-Tumor Cells’ KIR-HLA Interactions: “Missing Self”, “Induced Self” and Tolerance

**Figure 2:**
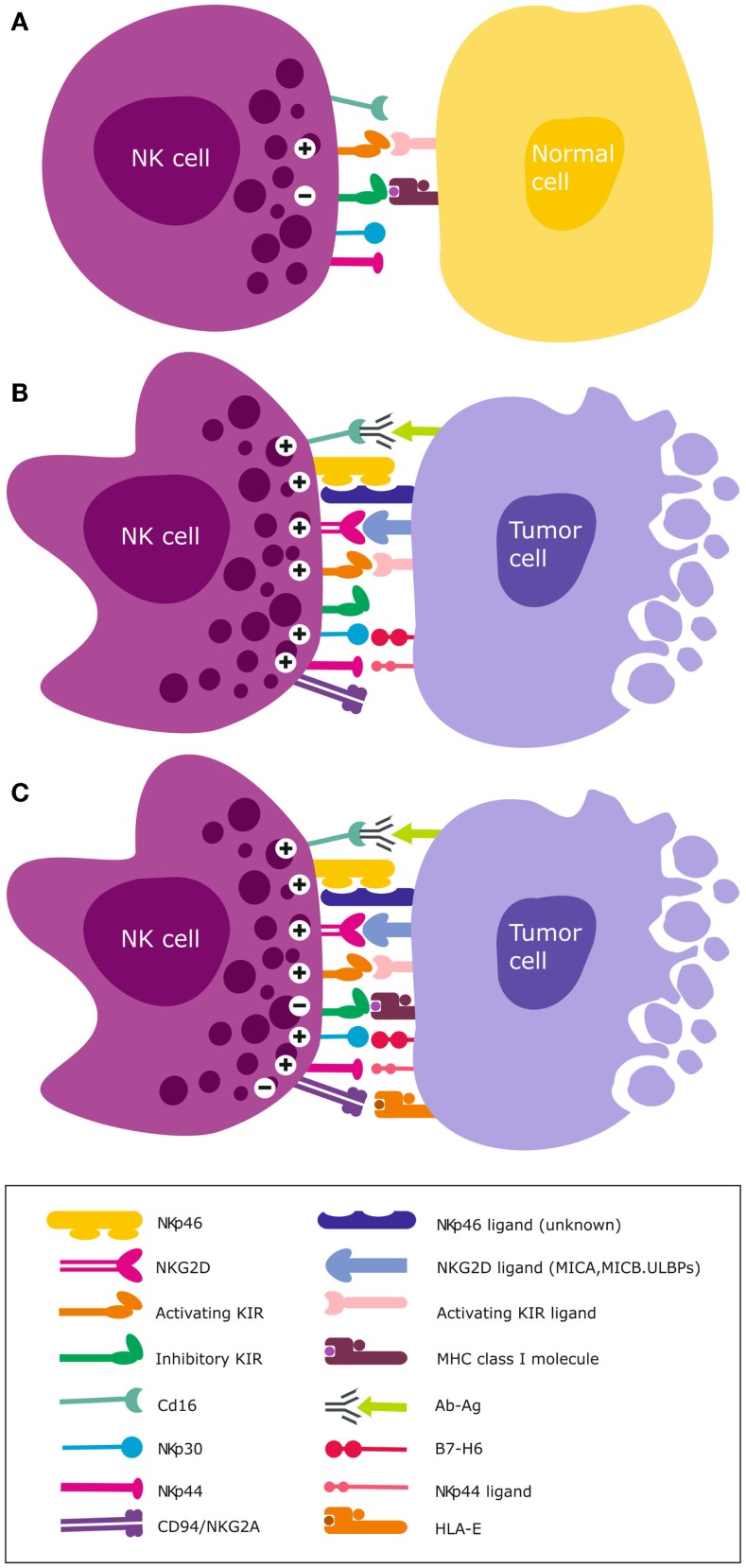
**NK cell activation and target recognition**. **(A)** *Tolerance to self*. NK cells express inhibitory and activating receptors on the surface and when interaction with cognate ligands for both activating and inhibitory receptors is balanced, NK cells remain tolerant and unable to kill the target cell e.g., normal unstressed self-cells that express class I HLA ligands for inhibitory receptors. **(B)** *Missing self*. A malignantly transformed cell may downregulate class I HLA but concomitantly express stress induced ligands that are recognized by NK cell activating receptors. A dominant activating signal is transduced that outweighs the inhibitory signals, resulting in NK cell activation and subsequent lysis of the target cell. **(C)** *Altered self*. Some cancer types express normal levels of class I HLA but concomitantly over-express stress-induced ligands e.g., GBM. Only when the activation signal transduced by ligation of stress ligands to cognate activating receptors overcomes the inhibitory signal, will NK cells become activated to lyse the target cell.

Natural killer cells express activating and inhibitory killer immunoglobulin-like receptor (KIR) genes encoded on chromosome 19q13.4 that are inherited and segregate separately from their ligands. These class 1 human leukocyte antigen (HLA)-A, -B, and -C are allelic variants that are encoded on chromosome 6p21.3. Mutations in the β2-microglobulin gene and loss of heterozygosity on chromosome 6 that harbors the class I and II HLA are common aberrations in cancer (162, 163). They invariably lead to downregulated expression of HLA molecules. Although the current review focuses on NK cells, in heterogeneous tissues KIRs are also expressed on CD8^+^ T cell subsets, albeit as a unique repertoire of single dominant KIR that impacts their function (164, 165). Through ligation of their KIRs to cognate HLA ligands on autologous cells, NK cells become educated to distinguish healthy self-cells (Figure 2A) from non-self cells that lack or possess poorly recognized polymorphic HLA ligands [deemed “missing self” (166); Figure 2B]. These malignant cells exhibiting HLA loss are thus recognized as missing self by educated NK cells, triggering their cytotoxicity (166, 167) (Figure 2B).

Educated NK cells can also distinguish healthy self-cells from altered-self cells that may express appropriate HLA ligands but concomitantly express stress-induced ligands (Figure 2C). Inhibitory KIRs are essential for this exquisite distinction of self from non-self cells and their main HLA ligand specificities are HLA-C. We recently demonstrated that although GBMs express high levels of class I HLA (39, 40), these malignant brain tumors may nevertheless become targets for NK cells due to the “altered self” mechanism (67, 72, 168) (Figure 2C). This is because the tumor cells concomitantly overexpress stress-induced ligands that are recognized by activating NKG2D receptors (39, 40, 169). Although GBM cells *proper* express low to negligent non-classical HLA-E, antigen presenting cells that constitute upto 50% of cells of the TME highly express HLA-E and may contribute to further tolerization of tumor infiltrating KIR^-^NKG2A^+^ NK cells (39, 40). Ligation of the self-HLA ligands to cognate inhibitory KIR results in phosphorylation of the ITIMs by Src family protein tyrosine kinases (PTKs). This creates docking sites for protein tyrosine phosphatases SHP-1 and SHP-2 (170) that inhibit NK cell activation by dephosphorylating proteins involved in downstream activation signaling. This inhibitory signaling maintains NK cells tolerant to normal self-cells. Ligation of activating KIRs transduces activating signals through phosphorylation of DAP12 or FcεRI-γ adaptor proteins containing immunoreceptor tyrosine-based activation motif (ITAM) by the Src PTKs (171). Downstream NK cell cytotoxicity pathways converge on the PI3K/Akt and mitogen-activated protein kinase (MAPK) pathways. The ensuing activating signal may override the inhibitory signals and ultimately, the overall threshold strength of activating or competing inhibitory signals determines whether the NK cell will be triggered for cytotoxicity (172, 173), cytokine release, or tolerance against encountered target cells.

However, only 5% of individuals possess all KIR genes in their genome and different individuals display highly variable KIR repertoires with up to 30,000 distinct NK cell subsets in peripheral blood (174). It is estimated that 10–20% autologous NK cells express inhibitory KIRs that do not recognize HLA-A, HLA-Bw4, or HLA-C self-alleles. These NK cells are considered “uneducated” and remain functionally tolerant against self-cells (175, 176). This is likely due to constitutively expressed CD94/NKG2A receptor that ligates non-classical HLA-E alleles to maintain tolerance, in complementation with KIRs to prevent NK cell autoreactivity (177, 178). This interpretation may be debated since uneducated cells should not express CD94/NKG2A since by definition this receptor provides NK cell education. However, there might be a qualitative difference in KIR-inhibition and NKG2A inhibition (179). NKG2A induces stronger cytokine responses and less degranulation than KIRs do. The NKG2A-inhibition may be easier to overcome and may be more important in microenvironments where HLA-expression is low. The ligand HLA-E is more commonly expressed by hematopoietic tumors but is less abundantly expressed by solid tumors, in particular GBM.

Other studies, however, demonstrated that NK cells that lack KIR for self-HLA, or lack self-HLA to cognate KIRs may instead be educated through CD94/NKG2A/HLA-E interactions. Functional activity manifests in transplant cases where donor NK cells express low frequencies of KIR or during the early phases after haploidentical hematopoietic stem cell transplantation (HSCT) (180, 181) when the donor NK cells’ KIRs have yet to reconstitute to pre-transplant levels. However, this might not be a general mechanism as it was also demonstrated that tolerance is maintained after HLA-matched sibling transplantation (182). Discrepancies between these two clinical studies may be due to emergence of conditions that influence endogenous cytokine levels. These include graft versus host disease (GvHD), bacterial infections or reactivation of human cytomegalovirus (CMV), as well as use of immunosuppressive drugs. Likewise, tolerance of uneducated NK cells may be abrogated by exogenously administered cytokines (179). These NK cells were subsequently shown to execute graft versus malignancy effects and improve survival after HSCT for AML/myelodysplastic syndrome (AML/MDS) (183). In an elegant study, neuroblastoma patients possessing one or more inhibitory KIRs missing self-HLA ligands (NS-KIRs) exhibited superior overall survival and progression free survival when treated with the anti-GD2 mAb 3F8 compared to patients possessing all KIRs in the presence of self-HLA (S-KIRs) following chemotherapy or autologous stem cell transplantation (141). This effect was attributed to the uneducated NK cells, even though *in vitro* experiments demonstrated that both educated and uneducated NK subpopulations were activated for ADCC by 3F8mAb. The difference between the *in vitro* and *in vivo* effects was attributed to increased IFN-γ release after ADCC that upregulated expression of self-HLA ligands and subsequently tolerized the educated NK cells expressing S-KIRs. It is also possible that other cellular subsets may have contributed to the inhibition signal of the effector cells in patients but not *in vitro*, such as γδT cells that also express CD94/NKG2A (184). In fact the interpretation can be further refined when considering that NK responses are not of “all or nothing” binary function. NK cell responses are functionally fine-tuned by the varying combinations of particular inhibitory KIRs with distinct affinities to cognate HLA ligands in different individuals formulated in the “rheostat” model (185, 186). However, the contribution of activating receptors has also been proposed (187). Furthermore, uneducated NK cells edit and eliminate immature DCs (iDCs) that express low levels HLA-E through contact dependent ligation of NKp30 (188), and thus enhance the adaptive response by potentiation of antigen presentation by mature DCs (Figure 1). Thus, uneducated NK cells play a significant role in shaping NK cell mediated tumor surveillance and treatment response.

## Clinical Basis of “Missing Self” Mechanism

The missing-self recognition (166, 189) has been exploited to the greatest extent clinically in the context of HSCT for treatment of leukemia (189). When HLA-matched related or unrelated donors possess NK cell subsets that express KIRs with specificity for particular class I HLA ligands that are missing in the recipient’s cancer, a receptor-ligand mismatch (190) between the donor’s inhibitory KIR and recipient’s HLA arises (KIR-HLA mismatch) (Figure 2B) (in allogeneic, transplant setting). This mechanism has been shown to induce potent NK cell alloreactivity and to mediate graft versus malignancy effect but not treatment limiting GvHD in some leukemia types (189, 191). Since NK cells are the first lymphocyte subset to recover following HSCT, it is likely that they mediate the early graft versus malignancy effects (192). However, the benefit of KIR-HLA mismatch in unrelated donor, allogeneic HSCT has been debated. Several contradicting studies reported increased transplant related mortality (193), reduced overall survival and progression-free-survival (194) due to infections, resulting in higher relapse rates. Discrepancies such as age and disease stage of the patient at the time of allograft, differences in extent of T cell depletion through, either antithymocyte globuline (ATG) use, myeloablative regimen, or source and number of CD34^+^ stem cells, may account for the conflicting benefits of KIR-HLA mismatch reported (191, 194). The KIR-HLA interaction models used to determine NK-mediated graft-versus-leukemia (GVL) may also generate different results [i.e., HLA-ligand mismatch (189); KIR mismatch (195); receptor-ligand mismatch (196) or missing ligand (183)]. Recently KIR allelic polymorphisms have been reported to impact the effect of KIR-HLA ligand interactions, for example, the 25 alleles of KIR2DL1 have been shown to display variable inhibitory strengths and duration of expression at the surface after interaction with HLA-ligands. Thus, donor grafts containing KIR alleles with appropriate polymorphism motifs are associated clinically with fewer relapses, less transplant related mortality, and improved survival (197) regardless of primary disease, T cell depletion, or donor type. Increased GVL can be demonstrated under some transplantation conditions pointing to the possibility that KIR-HLA interactions are important in tumor control.

## Activating KIRs and Haplotypes for Predicting NK Cell Potency Against Cancer

A study with mixed hematological malignancies reported that in the context of KIR-HLA ligand mismatch, donor haplotype B with high activating KIR genes (4–8 versus 1–3) was associated with poor disease-free survival (194), consistent with the disarming theory (198). However, they also analyzed impact of single KIR genes on relapse and revealed increased risk for AML, MDS, and CML patients transplanted with donors possessing KIR3DS1, KIR2DS1, and KIR2DS5. The latter two also display high linkage disequilibrium with KIR2DS3. Other studies demonstrated improved relapse-free and overall survival for AML patients with haplotype B (199), with the strongest prediction in the centromeric B region (Cen B) (200). Recently, haplotype B with telomeric A/B genotype, KIR2DL5, and KIR2DL1 in the presence of HLA-C2 strongly predicted post induction minimal residual disease with 100% sensitivity and 80% specificity in a cohort of 244 childhood lymphoblastic leukemia (201). We recently reported that NK cells isolated from healthy donors with haplotype B, KIR2DS2, and centromeric A/B or B/B, telomeric A/A or A/B genotype were the most potent against GBM cells *in vitro* and *in vivo* in mice compared to donors with KIR2DS4 (haplotype A, centromeric A/A, telomeric A/A) and KIR2DS2^-^/KIR2DS4^-^ negative donors (centromeric A/A, telomeric A/A, A/B, or B/B) (40). The KIR2DS2^+^ NK cell subsets constitutively elevated CD69 and CD16 activation markers that remained elevated when in contact with GBM target cells. The KIR2DS2^+^ NK cell subsets preferentially degranulated granzyme A from elevated LAMP-1^+^ cytolytic lysosomes (40). Others reported that KIR2DS1^+^ NK cells from HLA-C1 donors efficiently lysed target cells with HLA-C2 ligand *in vitro* (202), although this was possibly mediated through KIR-HLA mismatch. Another study with fewer patients demonstrated contradicting results of worse outcome (203). The presence of KIR3DS1 was reported to decrease relapse rates in Bw4^+^ recipients (204), diminish risk for acute GvHD, and increase overall survival (205, 206). These effects were potentiated in the presence of two copies of KIR3DS1 gene. From these cumulative studies, it appears both inhibitory and activating KIRs are important for anti-tumor responses, although qualitative differences in the composite KIRs defining the haplotypes may be better predictive of treatment response.

**Figure 3:**
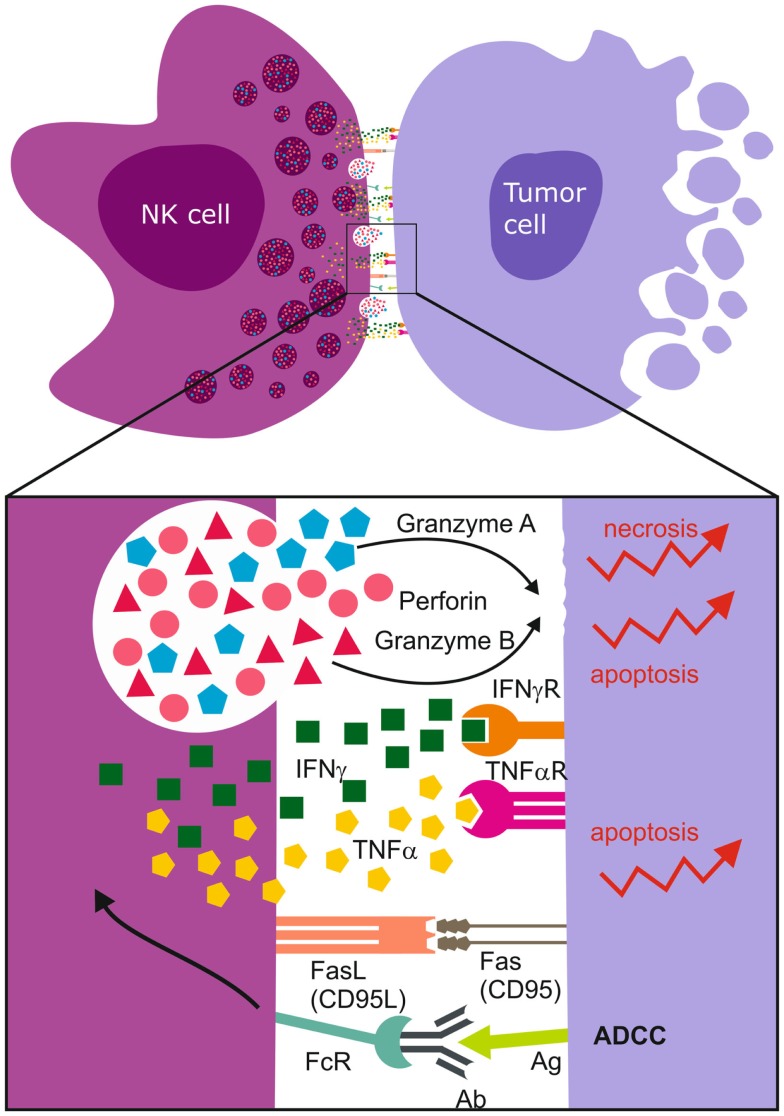
**Immunological synapse and NK cell mediated cell death**. Upon synapsing with target cells, NK cells release proteolytic enzymes, granzyme A and B, as well as perforins that induce necrotic death. The ligation of Fas Ligand (FasL) to Fas/CD95 receptor together with the binding of TNF-α to death receptors induce apoptosis of the tumor cell. NK cell FcγRIII (CD16) recognizes antibody Fc constant domains resulting in cytokine secretion and NK cell mediated antibody dependent cellular cytotoxicity (ADCC). The most abundantly secreted cytokines are TNF-α and IFN-γ.

Ultimately, cytotoxicity requires that the NK cell contacts its target cell and form a functional immunological synapse (207, 208) for optimal site directed secretion of perforin and granzymes A/B proteases (Figure 3). Intuitively, treatment response is also determined by the sensitivity of the cancer cells to the particular mode of death induced. Granzyme B induces rapid, intrinsic apoptosis mediated through cleavage of BH3-only pro-apoptotic protein Bid, cytochrome *c*, and downstream effector caspases 3 and 8 (209). Activated NK cells also express Fas Ligand [CD95L, (196), TNF-α, and TNF-related apoptosis inducing ligand (TRAIL)], and ligation of Fas (CD95) or TNF-α to death receptors leads to apoptosis of the receptor-bearing cell (67) (Figure 3). Although GBMs express the DR4, DR5 TNFR death domains, and CD95, they are inherently resistant to TRAIL and FasL. This is possibly due to deregulated downstream cascades involving BCL-2 and X-linked inhibitor of apoptosis proteins (XIAP) (147). Granzyme B also induces cytochrome *c* release by cleavage and inactivation of the anti-apoptotic BCL-2-family member Mcl-1 and substrates such as poly (ADP-ribose) polymerase (PARP), DNA-dependent protein kinase (DNA-PK), ICAD, and lamin B to generate double DNA-strand breaks. However, this signaling cascade is deregulated in solid cancers due to frequent p53 mutations, for example, in GBMs where p53 is mutated in 40% of cases (210). This results in upregulation of the pro-survival proteins, BCL-2 and BCL-X_L_, but downregulation of Bax, rendering them refractory to apoptosis (147). In contrast, granzyme A induced non-apoptotic cell death through release of ROS (211, 212), cleavage of nuclear lamins and histone H1 that are required for nuclear envelope stabilization and maintenance of chromatin structure (212, 213) to facilitate activity of DNAses. We recently demonstrated that potent alloreactive and highly activated KIR2DS2^+^ NK cell subsets killed apoptosis resistant GBM cells by preferentially degranulating granzyme A (40). Multiple mechanisms of inducing cell death are necessary to ensure the elimination of apoptosis-resistant cells (Figure 3).

## Clinical Trials Exploiting NK Cell Activity Against Cancer

### Autologous NK cell transfer

To circumvent the poor tropism into the tumor of high numbers of patients’ own NK cells, the latter can be expanded to high yields, activated *ex vivo*, and re-infused into patients’ circulation or tumor. This approach has the advantage of decreased risk for transplant related infections, graft rejection, or GvHD, lack of requirement for immunosuppression and histocompatibility matching. However, patients’ own educated NK cells might be inhibited to efficiently kill the tumor by self-HLA and the hostile immunosuppressiveTME. Autologous lymphokine-activated killer cells (LAK: polyclonal fractions of NK and T cells) were activated *ex vivo* in IL-2 and re-infused into melanoma and renal cell carcinoma patients, followed by adjuvant high dose IL-2 (214). The treatment was partially effective, although limited by low NK cell composition of the LAK product and severe toxicity due to IL-2 induced capillary leak syndrome. Furthermore, the adoptively transferred cells failed to persist *in vivo*. Although follow-up Phase II studies demonstrated that daily low dose (1.75 × 10^6^ IU/m^2^/day) subcutaneous IL-2 was better tolerated in relapsed non-Hodgkin’s lymphoma and breast cancer patients and expanded cytotoxic T cells, no improvement in patient outcomes was reported. Instead, the IL-2 treatment also expanded Tregs (217). One trial using recurrent malignant glioma patients’ own expanded and enriched NK cell fractions placed them in an Ommaya reservoir (215, 216, 218) in the resection cavity. The treatment was supplemented with 100 IU/kg IL-2 at implantation, followed by low dose IFN-β (6 × 10^6^ IU/week). The treatment was tolerated and partial radiological responses were reported (215). Attempts to circumvent the underlying tolerance administered a humanized IgG4 antibody IPH2101 that blocks KIR2DL1, KIR2DL2, and KIR2DL3 inhibitory signaling. However, as IPH2101 also binds KIR2DS1 and KIR2DS2, it may block crucial dominant activation signals provided by potent KIR2DS1, KIR2DS2 NK cell subsets (40). Indeed, IPH2101 has been investigated in early phase clinical trials for multiple myeloma and AML, and although few adverse events and toxicity were reported in multiple myeloma, long lasting objective responses were also not registered in the patients (219, 220), except for transient NK cell activation and cytotoxicity *ex vivo*. The next generation hinge-stabilized IPH2102 was recently shown to synergize with Lenalidomide to increase NK cytotoxicity of myeloma patients with FcγRIII and FcγRII polymorphisms treated with Daratumumab against CD38 (221). While IPH2102 blocked KIR-inhibitory signaling on the NK cells, Lenalidomide activated them to increase production of TNF-α, IFN-γ, and granzyme B. The tumor can also be sensitized to autologous NK cells by coadministering proteosome inhibitors (e.g., Bortezomib), doxorubicin, or histone deacetylase inhibitors that upregulate stress ligands for NKG2D receptors, induce pro-inflammatory cytokines or death receptors that bind TNF-α. Although Bortezomib was effective in preclinical models mediated by upregulated death receptors (TRAILR1-DR4, TNFR1, DR3 and DR6) as well as stress ligands on the tumor cells, it was not effective in AML and RCC patients (99, 222). The efficacy of Bortezomib might be contingent upon the inherent susceptibility of the tumor cell to undergo TRAIL and FASL dependent apoptosis. The dose, duration, and sequence of administration of agents when combining with NK cells requires fine-tuning as unexpected effects on NK cell function and or tumor sensitization may occur (223). During NK cell therapy, concurrent treatment with azacytidine and sorafenib, which impair PI3K and ERK phosphotylation might also hamper NK cell activity and be counter productive in augmenting tumor cell kill by the immunological mechanisms (224, 225). Lenalidomide was shown to enhance Rituximab induced ADCC against non-Hodgkin lymphoma and B-cell chronic lymphocytic leukemia through enhanced CD4^+^ T cell dependent IL-2 release, resulting in augmented NK cell activation, granzyme B, and FasL release (226). NK cells could also be stimulated to enhanced activation, proliferation, and persistence with anti-CD137 agonistic antibodies.

### Allogeneic NK cell transfer

Due to challenges posed by tolerance to self-cells, infusions of allogeneic NK cells derived from related or HLA-matched unrelated donors are increasingly pursued for adoptive cellular therapies. The cells can be obtained from donor bone marrow progenitors, umbilical cord, or peripheral blood leukapheresis product. Subsequently, T lymphocytes and B cells are depleted with anti-CD3 or anti-CD19 microbeads, respectively. This is essential to prevent GvHD and lymphoproliferative disorders (227) induced by transfer of T cells and Epstein Barr virus (EBV) transformed B cells, respectively. To prevent GvHD, the final T cell dose should be <3 × 10^5^ cells/kg or below 0.1% total lymphocyte count (99, 228). 1 × 10^8^ purified allogeneic NK cells have been safely transfused to patients without major toxicity, although a threshold of maximum tolerated doses have yet to be established. Initial studies demonstrated that absence of recipient lymphoid and myeloid cell depletion prior to donor cell infusion resulted in diminished NK cell persistence *in vivo* and reduced therapeutic efficacy. Thus, an immune suppressive “preparative” regimen became imperative prior to donor cell infusions. In a non-transplant treatment of AML patients, investigators compared low dose non-myeloablative fludarabine (25 mg/m^2^/day) for 5 days or high cyclophosphomide (60 mg/m^2^/day) for 2 days chased by fludarabine (25 mg/m^2^/day) for 5 days (Hi-Cy/Flu) prior to donor NK cell infusions and adjuvant IL-2 (1.75 million U/m^2^) for 14 days. Only patients receiving Hi-Cy/Flu regimen had *in vivo* detectable NK cells in peripheral blood 2 weeks post treatment based on PCR donor-recipient chimerism assay and measurable circulating IL-15 levels (99). Thus, depletion of the lymphoid and myeloid cells that also utilize and compete for IL-15 resulted in elevated serum levels of IL-15 and IL-7, allowing for homeostatic proliferation and expansion of the NK and CD8T cells *in vivo*. Objective therapeutic responses were correlated with the prolonged presence of donor NK cells in the recipient. The infused NK cells were tolerated and did not induce GvHD and notably, 25% high risk, poor prognosis AML patients exhibited complete responses (229). This regimen is widely used with minor modifications for trials in leukemia, non-Hodgkin’s lymphoma, breast, ovarian, and NSCLCs. Allogeneic NK cells combined with 2 weeks’ adjuvant IL-2 were administered in a phase II trial in 20 patients with solid tumors (14 ovarian, 6 breast cancer) following the low dose, non-myeloablative preparative regimen (230). Very few peripheral blood NK cells were detected after 1 week in the majority of patients and the expanded cells were mostly Tregs (99) due to the IL-2 treatment. Although proven safe in these settings, a major bottleneck to clinical efficacy remains consistent *in vivo* NK cell expansion and abrogation of the tumor induced immunosuppressive mechanisms.

## Methods for Expanding NK Cells in High Numbers

### Cytokines

Depending on whether a lymphodepletive regimen is given, typical NK cell infusions may vary from 8–20 × 10^6^ to 1 × 10^9^ cells/kg. In unconditioned patients, high NK cells doses are required to achieve therapeutic effects, thus high throughput expansion methods are required. Moreover, since NK cells constitute only 10–15% of peripheral blood lymphocytes, and freshly isolated cells are in resting state and not optimally cytotoxic, activation by cytokines in culture is additionally required. Initially, cytokine cocktails were administered *in vivo* but they evoked disappointing efficacy in clinical studies largely due to toxicity, or activation-induced cell death (AICD), or exhaustion (231) where the effector cells succumbed to apoptosis upon contact with vascular endothelium. Recently, cytokine therapy in preclinical studies demonstrated that IL-12, IL-18, and the genetically engineered “superkine” H9 reverted the functional anergy of NK cells that was induced by class I MHC deficient tumors and prolonged animal survival (232). NK cell stimulation with IL-12 and IL-18 elevated expression of T-cell immunoglobulin and mucin domain-containing (Tim)-3 receptor (233), previously associated with T cell exhaustion (234). However, in this study, TIM-3 was conversely proposed to denote NK cell activation and maturation, and only weakly suppressed NK cell cytotoxicity (233). “Memory” NK cells with enhanced proliferation and IFN-γ production could be generated by pre-activation with a cytokine cocktail including IL-12, IL-18, and IL-15 followed by a 3-week recovery period prior to re-stimulation with IL-12 + IL-15, or IL-12 + IL-18, or K562 (235). IL-2 increases NK cytotoxicity *in vitro* by increasing density of activation receptors (236), and sustains NK survival *in vivo* but off-target effects associated with vascular leak syndrome induced by stimulation of IL-2R on endothelial cells are problematic. The H9 superkine that signals independently of the IL-2Rα chain (237) may redress the above shortcoming and derive therapeutic benefits in the clinic. Combination IL-2 and IL-15 markedly prolongs NK cell survival. Uniquely, IL-15 inhibits IL-2 dependent AICD, (238) and decreases expression of PD-1. IL-15 promotes NK cell survival and expansion, especially when presented to NK cells *in trans* as a membrane bound complex with IL-15Rα by DCs and monocytes. Recently, a first in human trial investigated safety and maximum tolerated dose of a recombinant human (rh) IL-15 in patients with metastatic malignancy (239). Higher doses of IL-15 resulted in rapid redistribution of NK and CD8T cells from peripheral blood within 48 h followed by hyper-proliferation, where NK cells expanded upto 10-fold of baseline. The cells were highly activated and intensely released cytokines, IL-8, IL-10, TNF-α, IL-1β, IL-6, and IFN-γ, resulting in radiologically visible anti-tumor effects in some patients. However, the treatment was associated with dose limiting severe adverse events and the authors concluded the MTD for IL-15 in this format was 0.3 mg/kg/day (239). In other studies, CD34^+^ progenitor cells from umbilical cord blood (UCB) cells could be differentiated to NK cells in medium supplemented with low-dose GM-CSF, G-CSF, IL-6, and a high-dose cytokine cocktail consisting of IL-7, stem cell factor (SCF), IL-15, and IL-2. However, the cells displayed low CD16 and KIR expression at the end of the culture process (240, 241). Alternatively, CD34^+^ cells from adult peripheral blood could be differentiated in the presence of SCF, FLT3 ligand, IL-7, and hydrocortisone, followed by IL-7, IL-15, and hydrocortisone. No major toxicity was recorded when using these cells (240). These methods typically produce small-scale NK expansions in the range 10- to 20-fold that might not be sufficient to treat patients in trials.

### Feeder cells

Coculture of CD56^+^ purified NK cells with irradiated feeder cells has been demonstrated to expand and activate NK cells from T and B cell depleted peripheral blood of patients 800- to 1000-fold in a short time scale of 2 weeks (236). This NK cell product upregulated expression of NKG2D, NKG2C, secreted FasL, IFN-γ, IL-2, TRAIL, Granzymes A/B, and was highly cytotoxic against targets. The EBV transformed lymohoblastoid (EBV-LCL) feeder cells were irradiated with 100 Gy and cocultured in 500 IU/ml IL-2 at 20:1 ratio with 2 × 10^8^ purified peripheral blood NK cells obtained from 15 l leukapheresis product. Clinical grade NK cells expanded upto 3 × 10^10^ within a 3-week period (236). However, attempts to further expand beyond this failed, and it is not clear if this is due to AICD or replicative senescence due to shortened telomeres. Importantly, this study also demonstrated that previously expanded and frozen NK cells, when subsequently thawed, require further activation in culture with IL-2 to maintain their cytotoxicity although their viability is substantially compromised. Others have utilized feeder cell-free, automated bioreactor systems to expand highly cytotoxic cells from polyclonal PBMCs with 77-fold yield in 21 days. However, these were impure and consisted mostly T cells (CD3^+^CD56^-^) and NKT cells (CD56^+^CD3^+^) (242). This product had very low NK cells, average 38% (range 10–80%), and bears the treatment limiting risk of inducing GvHD. However, an autologous setting may be more permissive of T cell contamination, although they may differentiate into Tregs. Further manipulation to deplete the T cells prior to infusion in the patient would be required and is potentially costly (243). The K562 CML cells have been engineered to express membrane bound IL-15 gene fused to CD8α transmembrane domain as well as membrane bound 4-1BBL (mb15-41BBL) and shown to induce nearly 22-fold NK expansion within 1 week culture in 10 IU/ml IL-2 (244). In other studies, K562-based artificial antigen presenting cells were modified to express membrane bound IL-15 or IL-21 (245). Upon comparison, MbIL-21 expanded cells were superior to mbIL-15 expanded NK cells in that they exhibited an activated phenotype (denoted by elevated CD160 expression, (246), proliferated better, and elongated their telomeres (postulated due to STAT3 dependent signaling). They retained expression of KIR, CD16, produced cytokines, and were potent mediators of cellular cytotoxicity. Depending on the class I HLA ligands expressed on the surface of the feeder cells, it may be possible to selectively expand NK cells with particular KIR repertoires selected for potent efficacy (247). Since the alloreactive fraction in the polyclonal NK cell pool varies widely from 0 to 62% among potential donors (177), not all individuals are expected to be potent donors. Improving our understanding of molecular KIR/HLA combinations that predict best therapeutic benefit is imperative in next generation NK cell therapy. We recently demonstrated that donor NK cells with KIR2DS2 immunogenotype exhibited potent efficacy against GBM targets (40), even though they represented only 36% of the total NK cell pool. It is highly compelling to investigate whether selective expansion of these highly activated KIR2DS2 NK cell fractions would yield greater clinical benefits for GBM patients than using bulk NK cells.

## Conclusion and Perspectives

Our deeper understanding of NK cell biology and function in anti-cancer immunity, the increasingly precise methodologies for their characterization, purification, and expansion have all heightened our interest in exploring the applicability of NK cells for cancer immunotherapy. However, good manufacturing practice approved protocols for generating high quantities of pure, activated NK cells for repeated infusion into patients remain complicated, expensive, and are still somewhat experimental. Likewise, appropriate conditioning regimens to prevent graft rejection, that can be used for a wide range of cancer types, the right choice of exogenous cytokines to safely sustain homeostatic NK cell expansion, activation, and survival *in vivo* are all issues still under investigation. Nevertheless, concerted research efforts are underway to refine and renew the protocols to establish NK cells as efficacious effectors against malignancy. It is intriguing that the multitude of ways to enhance NK cytotoxicity seems to impact fundamental regulatory mechanisms such as receptor-ligand mismatch, expression of activating receptors, increasing receptor affinity, augmentation of ADCC. All the same, we do not yet know how to reap maximal benefit of this expertise in the clinic. It may be because the clearest evidence accrued so far is most often seen in larger cohort studies. Nevertheless, the salient question is what will it take to make NK cell therapy standard care for GBM and other solid cancers? Due to the poor tumor infiltration and limited passage across the blood brain barrier (39, 248), NK cell therapy in the brain might be best suited as a haplotype-matched, intralesional combination NK cell therapy with mAbs. Exploiting NK cell subsets with best predictive KIR/HLA combinations might be advantageous, since autologous NK cells previously demonstrated limited efficacy. For this to be effective, tumor reduction prior to NK therapy is an imperative in order to give the NK cells a task they can manage. The cells could be injected into and around the resection cavity and administered as an adjuvant therapy post standard treatment that combines debulking surgery, concomitant Temozolomide chemotherapy, and conformal ionizing radiation (249). This would exploit the period of a relative immune compromise (250) to promote homeostatic proliferation of grafted cells and prevent graft rejection. A phase I dose escalation toxicity study would be required as the brain may present unique toxicity and necessitates careful trial design. Strategies to consider might be single treatment with high numbers (effector: target ratios) of maximally activated cells or lower numbers and repeated doses to promote *in vivo* persistence in the recipient. We recently demonstrated in preclinical models that large, single NK cell doses were efficient and tolerated whereas repeated high doses were poorly tolerated in mice (40). If these results were translatable to humans, in a patient phase I trial, smaller dose escalations with maximally alloreactive subsets may be better tolerated, not least more feasible with regards to *ex vivo* NK cell expansions. Moreover, we demonstrated using multiparametric physiological magnetic resonance imaging (MRI) to identify predictive biomarkers, that 2 × 10^6^ intralesional NK cells were more effective compared to 1 × 10^6^ cells, even when these were combined with mAb9.2.27 in nude rats (158, 251). Ultimately, NK cells in solid tumors should be combined with mAbs against abundant tumor specific antigens to enhance their efficacy via ADCC and overcome inherent immunosuppression in the tumor bed (158). Immunologic surrogates that predict efficacy in patients could be IFN-γ, as this has been previously correlated with anti-tumor responses and overall survival (103).

## Conflict of Interest Statement

The authors declare that the research was conducted in the absence of any commercial or financial relationships that could be construed as a potential conflict of interest.
